# Targeting gut microbiota with short-chain fructo-oligosaccharides prebiotic fibers to support metabolic health in overweight prediabetic adults: a randomized, double-blinded, placebo-controlled study

**DOI:** 10.3389/fnut.2025.1718169

**Published:** 2025-12-17

**Authors:** Cindy Le Bourgot, Odile Capronnier, Sahara Graf, Thomas Carton

**Affiliations:** 1 Department of Scientific and Regulatory Affairs, TEREOS, Moussy-le-Vieux, France; 2 Department of Biometry, BIOFORTIS SAS, Saint-Herblain, France

**Keywords:** prebiotic, fructo-oligosaccharides, scFOS, gut microbiota, diabetes, metabolic health, weight management, randomized clinical trial

## Abstract

**Background and objectives:**

The global rise in metabolic disorders such as obesity and type 2 diabetes is largely driven by dietary changes and sedentary lifestyles. Prebiotic dietary fibers may help mitigate this trend by modulating gut microbiota. This study aimed to evaluate the effects of short-chain fructo-oligosaccharides (scFOS) on glucose homeostasis, body composition and gut microbiota in overweight individuals with prediabetes.

**Methods:**

In this randomized, double-blind, placebo-controlled, parallel arms trial, participants with fasting glycemia between 1 and 1.25 g/L and BMI between 23 and 35 kg/m^2^ received either 20 g/day of scFOS (Actilight^®^ 950P; Beghin-Meiji, France) or placebo for 12 weeks. Glucose metabolism, body composition and gut microbiota were assessed at baseline and post-intervention. The primary endpoint was the change in HbA1c level, with a hypothesis of superiority, tested using a linear mixed model.

**Results:**

Participants (*n* = 66, 35 scFOS, 31 placebo) had a mean age of 50.6 ± 9.0 years and BMI of 28.2 ± 2.7 kg/m^2^. Compliance was excellent (> 97%). No significant treatment effect was observed for glucose metabolism markers, including the primary outcome HbA1c (+0.055% with scFOS *vs* +0.030% with placebo, *p* = 0.6835). However, body composition outcomes favored scFOS: fat mass decreased (medians: −0.26% *vs* +0.20%, *p* = 0.0273), lean mass increased (+0.27% *vs* −0.30%; *p* = 0.0279). Body weight remained stable with scFOS while it tended to increase with placebo (estimated means: +0.14 kg *vs* +0.70 kg; global treatment effect: *p* = 0.0718). Among 30 participants analyzed for gut microbiota, *α*-diversity decreased in the scFOS arm and increased in placebo across three of four indices (*p* < 0.004), primarily driven by selective microbial shifts. In addition to a significant increase in *Bifidobacterium* (*p* = 0.0202), scFOS supplementation enriched *Anaerostipes*, while reducing *Blautia* and *Ruminococcus2* (*p* < 0.05). These changes were accompanied by increased fecal acetate (*p* = 0.0310) and propionate (*p* = 0.0062), contrasting with decreases in placebo.

**Conclusion:**

Twelve-week scFOS supplementation in overweight prediabetic adults led to beneficial changes in gut microbiota composition and fermentative activity, along with modest improvements in body composition. Although no significant improvements in glucose homeostasis were observed, this may reflect the moderately impaired metabolic status of participants and interindividual variability in response to prebiotics. Findings nonetheless suggest that scFOS may support gut and metabolic health and contribute to strategies for preventing or delaying metabolic disorders.

**Clinical trial registration:**

ClinicalTrials.gov, identifier NCT04767672.

## 1 Introduction

Over the past few decades, global dietary patterns have shifted markedly, with a growing intake of energy-dense, nutrient-poor foods. Combined with increasingly sedentary lifestyles, these changes have contributed to a dramatic rise in metabolic disorders, particularly obesity and type 2 diabetes (T2D). T2D is a chronic metabolic condition characterized by elevated blood glucose levels, which can lead to serious long-term complications. One of the main drivers of the increasing prevalence of T2D is the global rise in overweight and obesity, frequently associated with unhealthy lifestyle behaviors.

T2D typically develops gradually and is often preceded by a state of impaired glucose regulation, commonly referred to as prediabetes, in which fasting blood glucose levels range between 1.00 and 1.25 g/L. In Europe, approximately one in ten adults are estimated to have prediabetes (1) and are thus at high risk of progressing to overt diabetes. However, lifestyle interventions, particularly dietary modifications, have been shown to prevent or delay disease onset.

Recent research has highlighted gut microbiota as a potential contributor to the development of metabolic diseases. Indeed, several studies pointed out differences in intestinal microbiota composition and function between healthy people and patients with metabolic disorders, suggesting that dysbiosis, an imbalance in the composition yielding a non-optimal function of gut microbial communities, may play a role in blood glucose control and body composition (2–4). However, the eubiotic microbiota that supports healthy functioning can be obtained from different microbial ecosystems and cannot be attributed to a specific composition in healthy subjects. Similarly, the taxonomic characteristics of dysbiotic microbiota cannot be properly described. Among the factors shaping gut microbiota, diet is considered the most influential. In particular, dietary fibers with prebiotic properties have been shown to beneficially modulate the gut microbiota and improve metabolic outcomes (5, 6). Prebiotics are defined as substrates selectively utilized by host microorganisms that confer a health benefit (7). Their fermentation by gut microbes leads to the production of short-chain fatty acids (SCFAs), which are associated with improved glycemic control, lipid metabolism, and body composition (8).

Among the main prebiotic dietary fibers, short-chain fructo-oligosaccharides (scFOS) are well-studied for their selective fermentability and health benefits. They are produced by enzymatic synthesis from sucrose derived from sugar beet. They consist of a terminal glucose molecule linked to fructose units via β(1–2) bonds and have a low degree of polymerization (DP 3–5). In contrast, inulin is primarily extracted from chicory roots and has a broader DP range (2 to 60), while oligofructose is obtained by partial enzymatic hydrolysis of inulin and typically has a DP of 2 to 9. These structural differences influence their fermentability and their specific effects on the gut microbiota, with scFOS being more rapidly and selectively fermented by beneficial bacteria such as Bifidobacteria.

ScFOS are commonly used to enrich foods with fiber, reduce sugar content, and lower postprandial glycemic responses and energy density (9, 10). A systematic review and meta-analysis of animal studies reported significant reductions in fasting glycemia following FOS intake, particularly in models of impaired glucose homeostasis or presenting obesity (11). Despite promising preclinical findings, clinical evidence on the long-term metabolic effects of scFOS in at-risk populations remains limited and inconclusive (12–14). Clinical trials with other prebiotic fibers such as inulin and oligofructose have yielded inconsistent results, with outcomes varying depending on the population studied, dietary context, type and dose of prebiotic, and intervention duration (15, 16).

This study is among the few to simultaneously assess the effects of scFOS over a 12-week period on gut microbiota composition, glucose homeostasis, and body composition in individuals who are not yet clinically diabetic or obese, but who present a high-risk metabolic profile due to overweight and prediabetes. This integrative approach allows for a more comprehensive understanding of the potential of this early nutritional intervention to prevent the progression toward metabolic disease.

## 2 Materials and methods

### 2.1 Study design and subjects

A randomized, double-blind, multicenter, placebo-controlled, parallel-arm trial was designed to investigate the effects of a scFOS in overweight prediabetic participants and was performed between May 2021 and March 2024. Participants were volunteers recruited in the community and included by three clinical investigation units located in France.

To be eligible to the study, male and female volunteers had to fulfil the following criteria: (1) Age from 18 to 65 years; (2) 23 ≤ body mass index (BMI) ≤ 34.9 kg/m^2^; (3) Dysglycemic or prediabetic participants (i.e., fasting venous glycemia ≥ 1 g/L and ≤ 1.25 g/L at the inclusion visit V1) with no antidiabetic medication; (4) Consuming 10 to 20 g of fiber per day. Participants’ eligibility was assessed during V1 and confirmed during the randomization visit (V2), notably regarding their glycemic status and their fiber consumption (according to a 3-day food diary).

The main exclusion criteria included suffering from a metabolic, severe chronic or gastro-intestinal disorders, with a known or a suspected food allergy or intolerance or hypersensitivity to any food ingredient, taking or having stopped less than 3 months before V1 any treatment which could significantly affect assessment criteria according to the investigator (including dietary supplements, pro- and prebiotics, and antibiotics), with a significant change in food habits or in physical activity in the past 3 months, with a specific diet (hyper or hypocaloric, vegan, vegetarian…), with a personal history of eating disorders, consuming more than 2–3 drinks of alcoholic beverages daily or smoking more than 10 cigarettes per week. Participants were excluded if biological analyses performed on the blood sample at V1 was out of range: fasting blood triglycerides (TG) > 3.5 g/L, total cholesterol (TC) > 4.5 g/L or high-density lipoprotein cholesterol (HDLc) < 0.1 g/L, aspartate aminotransferase (ASAT), alanine aminotransferase (ALAT) or *γ*-glutamyltransferase (GGT) > 3 times the laboratory upper limit of normality, urea > 12 mmol/L or serum creatinine > 125 μmol/L, hemoglobin < 11 g/L, leucocytes < 3,000/mm^3^ or 16,000/mm^3^.

This study was approved by an Independent Ethics Committee named Comité de Protection des Personnes OUEST III (Poitiers, France), and the French Health Authority (ANSM) was notified of this study approval. A signed informed consent form was acquired from each participant before randomization. The trial protocol was registered at ClinicalTrials.gov as NCT04767672 on 22 February 2021.^1^

### 2.2 Randomization and blinding

During the randomization visit (V2), each subject was randomly assigned to either active product (scFOS) or placebo in accordance with the blocked randomization table generated using SAS^®^ software (version 9.4, SAS Institute Inc., Cary, NJ, USA).

The product allocation was defined using a dynamic randomization algorithm, designed to minimize imbalance between the 2 arms within the strata defined by centers and fiber intake (10–15 g and 15–20 g). The random product attribution was performed after checking the eligibility of the subjects once the results were available for inclusion, thus minimizing the selection bias. The investigator allocated the product through the randomization management interface (Interactive Web Response System), with no access to the random allocation sequence.

Researchers and participants were blinded to intervention assignments until the study was completed. The active product and the placebo were formulated as powder and had identical administration regimen. The packaging and labelling of both products were identical.

### 2.3 Intervention and procedures

**Figure 1:**
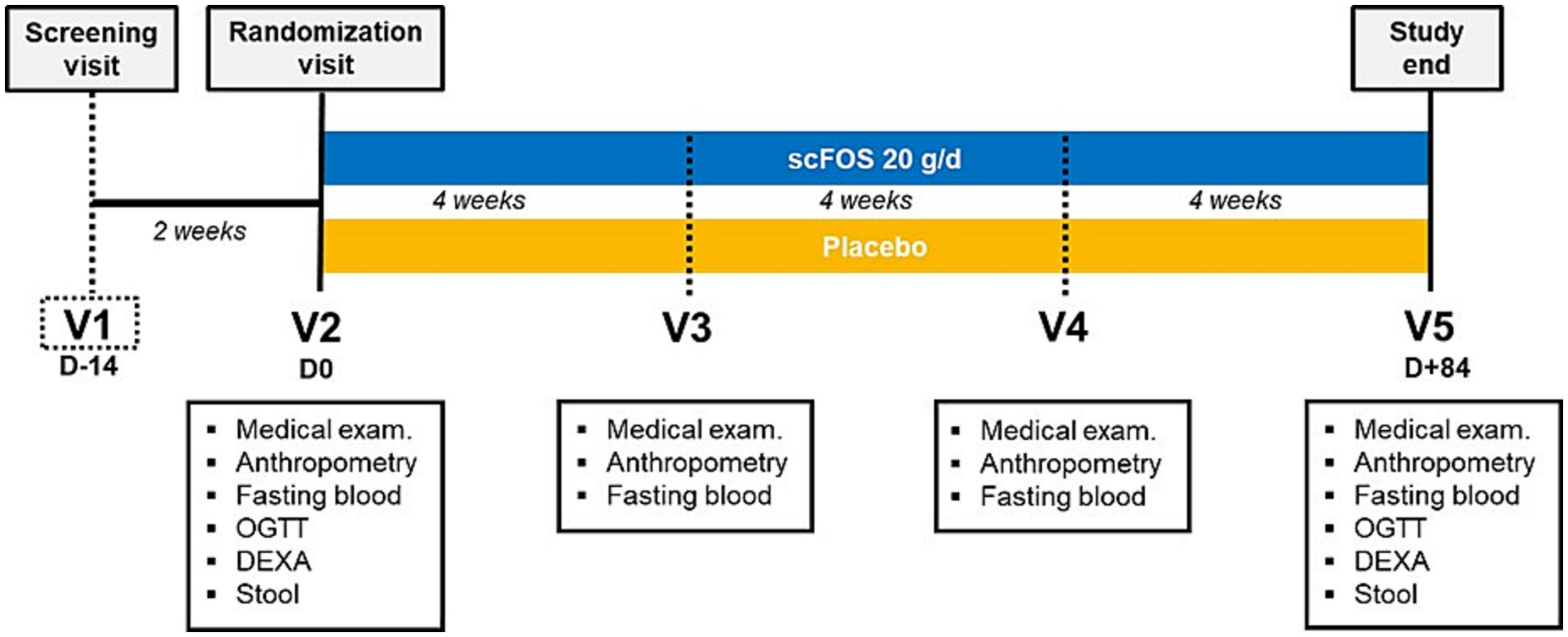
Study design. OGTT, oral glucose tolerance test; DEXA, dual-energy X-ray absorptiometry; scFOS, short-chain fructo-oligosaccharides.

From randomization and treatment start (V2), all participants underwent different medical examinations and data collection at several time points for 3 months (Figure 1) including anthropometric measurements (weight, height, waist and hip circumference), blood sampling and stool sample collection at home. Additionally, assessment of body composition via dual-energy X-ray absorptiometry (DEXA) and an oral glucose tolerance test (OGTT) were performed at the start (V2) and end (V5) of the 12-week supplementation. Dietary intake was recorded via a 3-day food diary (paper form), self-completed at home before each visit (V2 to V5). Participants were instructed by a dietetician how to record all their food intakes over the 3-day period. Data were then analyzed with Nutrilog^®^ software (Marans, France) to calculate the mean daily intake of total energy (kcal/day), macronutrient (g/day) and fiber (g/day). These measures were used to monitor dietary habits and detect any major changes that would have occurred during the intervention.

During the intervention period, participants were instructed to consume orally two 10 g sachets of prebiotic per day (Actilight^®^; 95% scFOS; Beghin-Meiji, France) or two 10 g sachets of placebo (maltodextrin; Tereos, France), one at breakfast and one at lunch or dinner.

The prebiotic scFOS was obtained from sugar beet sucrose through an enzymatic reaction. It is composed of a terminal glucose molecule (G) linked to fructose molecules (F) by a β1–2 bound, with 37 ± 6% 1-kestose (GF2), 47 ± 6% nystose (GF3), and 16 ± 6% 1F-β-fructofuranosyl nystose (GF4), and therefore presents a low degree of polymerization comprised between 3 and 5.

Participants were requested to maintain their regular diet and exercise routine. Physical activity was qualitatively monitored qualitatively at each visit (every 4 weeks during the 3-month period) via two multiple choice questions about the average duration of moderate and vigorous physical activity. In addition, participants were instructed to refrain from taking any antibiotics and other prebiotics, probiotics or synbiotics, or any other products that can directly impact the gut area. Any adverse events or discomfort experienced by the participants were to be noted down.

### 2.4 Blood parameters

Blood samples were collected under fasting conditions at four visits: baseline (V2, day 0, before treatment start), after 4 weeks of supplementation (V3), after 8 weeks of supplementation (V4), and at the end of supplementation (V5, 12 weeks after baseline) (see Figure 1). At each visit, HbA1c, glycemia, insulinemia, fructosamine, and safety biomarkers (alanine aminotransferase, aspartate aminotransferase, *γ*-glutamyltransferase, urea, serum creatinine) were analyzed using COBAS e411 and Integra400 + automated analyzers (Roche Diagnostics). At V2 and V5 only, active GLP-1 was analyzed (V-plex PLUS kit, MSD) and an OGTT was performed to measure glycemia and insulinemia 0, 60 and 120 min after ingestion of 75 g of glucose, after fasting overnight.

### 2.5 Fecal parameters

Stool samples were collected at most 48 h before V2 and V5 on a subpopulation of participants, to analyze fecal bacteria composition and diversity by 16S metabarcoding, and to measure SCFA levels. Stools were collected at home and kept between 2 and 8 °C in a cooler until returned to the investigator at the next visit. They were then aliquoted and stored at −80 °C for further analysis at the central laboratory.

Bacterial DNA was isolated from stool samples by magnetic capture in a Maxwell16 instrument (Promega) after mechanical lysis of the fecal sample on a FastPrep-24 (MP Biomedicals) and the variable regions 3 and 4 of the 16S rRNA gene were amplified (17) and paired-end sequenced by next generation sequencing (Illumina MiSeq) as previously described (18). The targeted metagenomic sequences were analyzed using a bioinformatic pipeline developed by Biofortis, based on Dadaist2 software v1.2.5 s (19). The barcoded paired reads were demultiplexed into single read sequences, which were then paired into longer fragments and cleaned. After quality-filtering and sequencing error modeling, amplicon variant sequences (ASVs) were obtained. A taxonomic assignment of these ASVs was performed to determine bacterial community profiles.

Fermentative activity of the fecal microbiota was assessed by measuring SCFA concentrations (acetate, propionate, butyrate, isobutyrate and valerate) using gas chromatography coupled with mass spectroscopy and calculating total SCFAs.

### 2.6 Outcomes

The primary outcome of the study was the absolute variation of HbA1c level between V2 and V5. Other parameters related to glucose metabolism were studied as secondary endpoints. The homeostasis model assessment of insulin resistance score (HOMA-IR) was calculated as cited in Wallace et al. (20) according to a transposition of Matthews’s original equation (21). The quantitative insulin sensitivity check index (QUICKI) was calculated according to Katz’s equation (22). The insulin sensitivity index (ISI) proposed by Matsuda and Defronzo (23, 24) was derived from OGTT data obtained at T0, T60 and T120. The incremental Area Under the Curve (iAUC) between T0 and T120 of glycemia (g/L.min) and insulinemia (mU/L.min) during the OGTT were also computed and change from V2 to V5 was analyzed.

Other secondary endpoints were change from V2 to V5 in gut microbiota composition (relative abundances at the phylum, family and genus levels), *α*-diversity indices (observed richness, inverse Simpson, Shannon and phylogenetic diversity [PD]), *β*-diversity dissimilarity indices (Bray-Curtis, Jaccard and weighted UniFrac), fermentative activity (SCFAs), anthropometric parameters and body composition (bone mineral mass, total lean mass, total fat mass, bone mineral density). Safety was assessed at each visit from V2 to V5 through the systematic monitoring of vital signs, physical findings and blood biomarkers. In addition, the investigator questioned the participant at each visit to record any adverse events.

### 2.7 Statistical analysis

All statistical analyses were performed on the intention-to treat population (ITT; all randomized participants) and per-protocol population (PP; participants from the ITT without major protocol deviations). The results presented in this article primarily concern the ITT population, analyses on the PP population were supportive. Missing data were not replaced.

The primary endpoint was the absolute change of HbA1c level (%) between V2 (treatment start) and V5 (treatment end, i.e., after 12 weeks of consumption), tested with a hypothesis of superiority (greater reduction with scFOS). To detect a mean reduction of HbA1c from baseline of at least 0.8% after a 12-week supplementation with scFOS (standard deviation [SD]: 0.94%) compared to placebo (SD: 1.09%) as found in the literature (25), with a two-sided 5% significance level and a power of 85%, a sample size of 35 patients per arm was necessary, given an anticipated dropout rate of 10%. In the end, a total of 66 participants were randomized.

It was planned to analyze all changes from baseline using linear mixed models for repeated measurements with fixed effects of treatment, time (visit or timepoint according to endpoint), treatment-by-time interaction, baseline of the parameter of interest, and random effects on center (the latter being removed in case of non-convergence) and subject (when applicable). Abundance endpoints were log-transformed by default, as were other endpoints if the normality and homoscedasticity assumptions on residuals from raw data were not met graphically (i.e., fasting insulinemia, HOMA-IR, glycemia, insulinemia, total SCFAs). If the assumptions on both raw and log-transformed data were not met, the Wilcoxon rank sum test was applied to the variations at each visit (i.e., QUICKI, ISI-M, GLP-1, iAUC of glycemia, iAUC of insulinemia, acetate, propionate, butyrate, waist circumference, hip circumference, bone mineral composition, whole lean body mass, whole fat body mass, bone mineral density, total body mass).

For all statistical tests, the 0.05 level of significance was used to declare a statistically significant effect. A Bonferroni-Holm adjustment was used to correct for the multiplicity of comparisons at each visit / timepoint, for all secondary endpoints, when applicable.

Statistical analysis of clinical data was performed using SAS software version 9.4 (SAS Institute Inc., Cary, NC, USA), while microbiome data was analyzed using R version 4.3.3 (R Core Team, Vienna, Austria).

## 3 Results

### 3.1 Characteristics of participants

**Figure 2:**
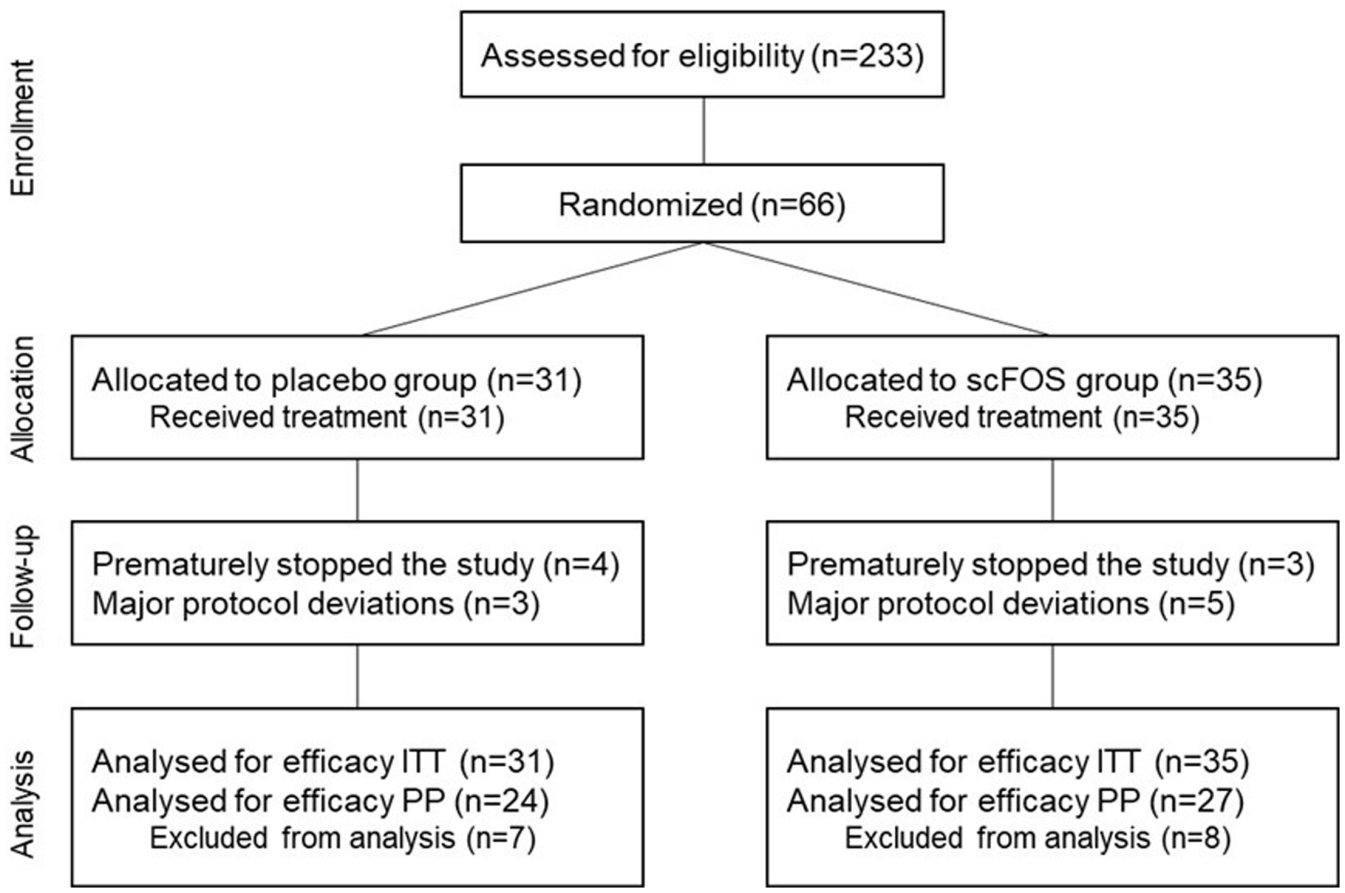
Study flow chart. ITT, intention to treat; PP, per protocol; scFOS: short-chain fructo-oligosaccharides.

A total of 233 participants were screened for this study, out of which 66 subjects were randomized based on the inclusion criteria (ITT population). The subjects were randomly allocated into a scFOS arm (*n* = 35) or placebo arm (*n* = 31). The subgroup analyzed for fecal parameters was made of 30 subjects (15 in each arm). Overall, 15 participants from the ITT population presented major deviations to the protocol and were therefore excluded from the PP population (*n* = 51). Indeed, 7 participants prematurely stopped the study before the end of treatment (V5) and 8 participants finished the study but were excluded after study completion primarily due to the use of concomitant treatments during the study period (Figure 2).

No meaningful difference was observed between the ITT and PP populations at baseline. Main baseline parameters and lifestyle characteristics of the ITT population by treatment arm are presented in Table 1, being quite similar between randomization arms.

**Table 1:** Descriptive baseline characteristics per study arm in the ITT population.

Variable	Placebo (*n* = 31)	scFOS (*n* = 35)
Sex		
Male, n (%)	17 (54.8)	21 (60.0)
Female, n (%)	14 (45.2)	14 (40.0)
Age (years)		
N	31	35
Mean (SD)	49.3 (8.2)	51.7 (9.5)
Median (Q1; Q3)	51.0 (44.0; 56.0)	54.0 (47.0; 58.0)
BMI (kg/m^2^)		
N	31	35
Mean (SD)	28.4 (2.8)	28.1 (2.6)
Median (Q1; Q3)	27.8 (25.9; 31.1)	27.8 (26.4; 29.5)
HbA1c (%)		
N	31	35
Mean (SD)	5.6 (0.4)	5.6 (0.4)
Median (Q1; Q3)	5.6 (5.4; 5.7)	5.5 (5.3; 5.8)
Fasting blood glucose (g/L)		
N	31	34
Mean (SD)	1.1 (0.1)	1.1 (0.1)
Median (Q1; Q3)	1.1 (1.0; 1.2)	1.1 (1.0; 1.1)
Dietary fiber intake (g/day)		
N	31	35
Mean (SD)	16.7 (3.0)	16.2 (4.8)
Median (Q1; Q3)	16.9 (14.4; 19.5)	16.2 (12.6; 19.3)

BMI, body mass index; HbA1c, glycated hemoglobin A1c; ITT, intention to treat; SD, standard deviation; Q1, 1st quartile; Q3, 3rd quartile.

The mean age of participants was 50.6 ± 9.0 years, with a mean BMI of 28.2 ± 2.7 kg/m^2^ corresponding to overweight.

Compliance was high in both treatment arms and at all visits (mean ratio of effective / theoretical sachets intake > 97%).

### 3.2 Glucose metabolism

No significant difference was observed between scFOS and placebo in the change of HbA1c from V2 to V5 (+0.055% *vs* +0.030%, *p* = 0.6835) in the ITT population. The analysis performed on the PP population led to the same conclusion (*p* = 0.7308). No statistically significant differences were observed in the other measured parameters related to glucose metabolism (Table 2). No overall product effect was observed when including the intermediate values obtained at V3 and V4 for all measured parameters. Similarly, no significant difference between arms was found for any OGTT parameter variations from baseline (glycemia and insulinemia at T0, T60, and T120 min, iAUC_0-120_ of glycemia and insulinemia) (data not shown).

**Table 2:** Absolute change from V2 to V5 of metabolic parameters per treatment arm in the ITT population.

Variable	Placebo (*n* = 31)	scFOS (*n* = 35)	scFOS vs. Placebo	*P*-value
HbA1c (%)*				
N	28	32		
Mean [95%CI]	+0.030 [−0.057; 0.117]	+0.055 [−0.027; 0.137]	+0.025 [−0.095; 0.144]	0.6835
Fructosamine (μmol/L)*^§^				
N	28	32		
Mean [95%CI]	−0.159 [−5.455; 5.138]	−3.888 [−8.877; 1.101]	−3.729 [−11.010; 3.551]	0.8930
Fasting glycemia (g/L)*^§^				
N	27	30		
Mean [95%CI]	+0.029 [−0.009; 0.067]	−0.027 [−0.063; 0.009]	−0.057 [−0.109; −0.004]	0.1019
Fasting insulinemia (mUI/L)*†^§^				
N	28	32		
Mean [95%CI]	+0.036 [−0.046; 0.119]	−0.013 [−0.091; 0.065]	−0.050 [−0.133; 0.033]	0.7131
HOMA-IR (a.u.)*†^§^				
N	27	30		
Mean [95%CI]	+0.040 [−0.052; 0.132]	−0.027 [−0.114; 0.059]	−0.067 [−0.161; 0.027]	0.4830
QUICKI (a.u.)^# §^				
N	27	30		
Median (Q1; Q3)	−0.005 (−0.015; 0.004)	+0.002 (−0.009; 0.018)		0.2018
ISI-M (a.u.)^#^				
N	24	30		
Median (Q1; Q3)	−0.100 (−0.790; 0.300)	+0.080 (−0.562; 0.854)		0.2470
Active GLP-1 (pM)^#^				
N	27	32		
Median (Q1; Q3)	−0.03 (−0.16; 0.12)	+0.01 (−0.13; 0.16)		0.3979

* Values are estimated means from the linear mixed model and their 95% confidence interval [95%CI] of the difference V5—V2 in each group, along with the between-group difference in estimated means and 95%CI of the difference, with the between-arm *p*-value. † Values were log-transformed for analysis. # Values are observed median, 1st (Q1) and 3rd (Q3) quartiles, with the between-arm *p*-value obtained from the Wilcoxon-rank sum test. § *p*-values adjusted due to the multiplicity of comparisons (multiple visits; Bonferroni-Holm method). HbA1c: glycated hemoglobin A1c; ITT: intention to treat; HOMA-IR: Homeostasis model assessment of insulin resistance score; QUICKI: Quantitative insulin sensitivity check index; ISI: insulin sensitivity index; GLP-1: glucagon-like peptide 1.

### 3.3 Body composition

Product effect on the changes in body weight and BMI from V2 to V5 was close to significance level (in the ITT and the PP populations for body weight, in the PP population for BMI; *p* < 0.1): body weight and BMI tended to increase in the placebo arm, while remaining relatively stable in the scFOS arm (Table 3).

**Table 3:** Absolute change from V2 of body weight and body mass index per treatment arm in the ITT population.

Variable	Visit	Placebo (*n* = 31)	scFOS (*n* = 35)	scFOS vs. Placebo	*P*-value	*P*-value*
Weight (kg)						0.0718
N	V3	30	33			
Mean [95%CI]		+0.35 [−0.17; 0.88]	−0.11 [−0.62; 0.39]	−0.46 [−1.19; 0.26]	0.2605	
N	V4	27	32			
Mean [95%CI]		+0.49 [−0.05; 1.03]	−0.25 [−0.76; 0.26]	−0.74 [−1.48; −0.001]	0.1489	
N	V5	28	32			
Mean [95%CI]		+0.70 [0.17; 1.24]	+0.14 [−0.37; 0.64]	−0.57 [−1.30; 0.17]	0.2605	
BMI (kg/m^2^)						0.1035
N	V3	30	33			
Mean [95%CI]		+0.11 [−0.07; 0.28]	−0.03 [−0.19; 0.14]	−0.13 [−0.37; 0.11]	0.3780	
N	V4	27	32			
Mean [95%CI]		+0.16 [−0.02; 0.34]	−0.08 [−0.24; 0.09]	−0.23 [−0.48; 0.01]	0.1824	
N	V5	28	32			
Mean [95%CI]		+0.22 [0.05; 0.40]	+0.06 [−0.11; 0.23]	−0.16 [−0.41; 0.08]	0.3780	

Values are estimated means from the linear mixed model and the 95% confidence interval (95%CI) of the difference Vn—V2 in each group, along with the between-group difference in estimated means and 95%CI of the difference, with the *p*-values for the between-group comparisons. *P*-values were adjusted due to the multiplicity of measurements (multiple visits; Bonferroni-Holm method). BMI: body mass index. *p-value for the global treatment effect.

In addition, some parameters of body composition as measured by DEXA displayed significant differences between treatment arms at the end of the follow-up. Body fat mass decreased in the scFOS arm while it increased in the placebo arm between V2 and V5. Conversely, lean mass followed the opposite trend. The treatment effect was statistically significant for absolute and relative changes in fat mass, and for the relative change in lean mass (Table 4).

**Table 4:** Absolute change from V2 to V5 of body composition parameters measured by DEXA per treatment arm in the ITT population.

Variable	Placebo (*n* = 31)	scFOS (*n* = 35)	*P*-value
Lean body mass (kg)			
N	27	32	
Median (Q1; Q3)	−0.19 (−1.00; 0.83)	+0.36 (−0.39; 0.93)	0.1781
Lean body mass (%)			
N	27	32	
Median (Q1; Q3)	−0.30 (−1.17; 0.60)	+0.27 (−0.19; 0.72)	**0.0279**
Fat body mass (kg)			
N	27	32	
Median (Q1; Q3)	+0.27 (−0.53; 0.86)	−0.15 (−0.97; 0.38)	**0.0400**
Fat body mass (%)			
N	27	32	
Median (Q1; Q3)	0.20 (−0.60; 1.17)	−0.26 (−0.72; 0.19)	**0.0273**

Values are observed median, 1st (Q1) and 3rd (Q3) quartiles, with the between-arm *p*-value obtained from the Wilcoxon-rank sum test. ITT: intention to treat; DEXA: dual-energy X-ray absorptiometry. Bold values indicate statistically significant differences between arms (*p* < 0.05).

Besides, although some variations in total energy, macronutrients and fiber intake from the diet were observed, no statistically significant between-arm differences were reported (Supplementary Table 1).

### 3.4 Gut microbiota: composition and fermentative activity

**Figure 3:**
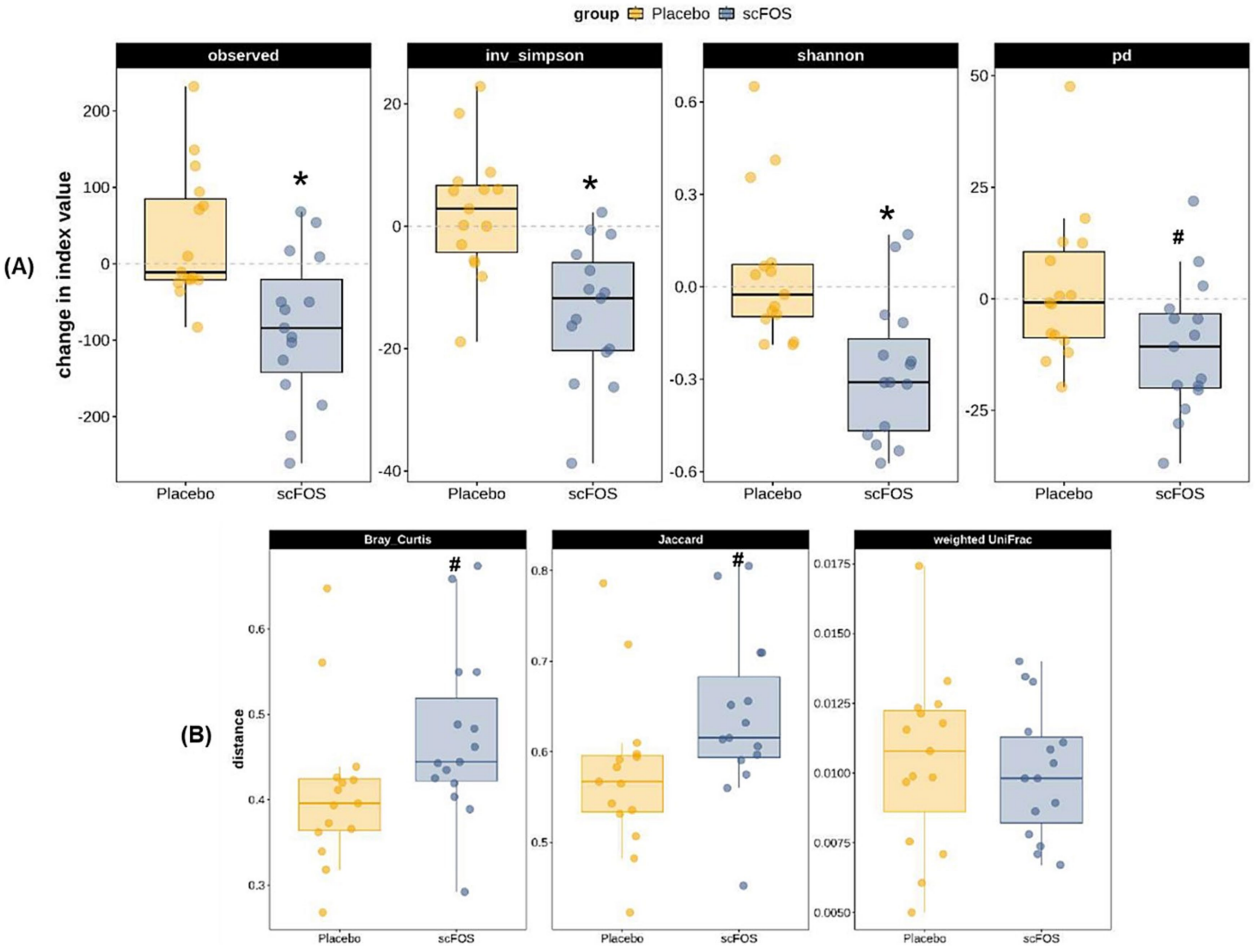
Absolute change from V2 to V5 of *α*-diversity **(A)** and β-diversity **(B)** indices for gut microbiota composition in the ITT population. Observed, observed richness index; inv. simpson, inverse Simpson index; shannon, Shannon index; pd., phylogenetic diversity index; Bray Curtis, Bray–Curtis dissimilarity index; Jaccard, Jaccard dissimilarity index; Weighted UniFrac, weighted UniFrac dissimilarity index. * *p* ≤ 0.05: significant difference between groups; # 0.05 < *p* < 0.10: trending difference between groups.

Regarding gut microbiota analyzed in a subpopulation of 30 participants, absolute changes of 3 *α*-diversity indices out of the 4 were statistically significant: Observed Richness, Inverse Simpson and Shannon indices decreased between V2 and V5 in the scFOS arm (*p* < 0.004; Figure 3A). The decrease in Phylogenetic Diversity was also close to significance (*p* = 0.0824), whereas an unsignificant slight increase was observed in the placebo arm for Observed Richness and Inverse Simpson. No statistically significant between-arm differences were observed for any of the three *β*-diversity dissimilarity indices in the ITT population. However, the difference in the change from V2 to V5 of taxonomic β-diversity was close to significance level when analyzed through Bray-Curtis (*p* = 0.0758) and Jaccard (*p* = 0.0663), the increase of taxonomic β-diversity tending to be higher in the scFOS arm compared to the placebo arm (Figure 3B).

Table 5 presents estimated changes in relative abundance of taxa from V2 to V5 in each treatment arm. The within-arm *p*-value refers to the difference between V2 and V5 in each arm, and the between-arm p-value refers to the difference of change at V5 between scFOS and placebo, which corresponds to the treatment effect. The most important increases observed with scFOS concerned the phylum *Actinobacteria*, and its children’s taxa: *Bifidobacteriaceae* family and *Bifidobacterium* genus; variations were > + 6% and were significantly higher than the changes observed in the placebo arm. The *Anaerostipes* genus also significantly increased under scFOS (+4.94%), significantly more than under placebo, while its parent family (*Lachnospiraceae*) and phylum (*Bacillota*) were not significantly impacted. Conversely, the sibling genus *Blautia* markedly decreased in the scFOS arm (−8.46%), significantly more than in the placebo arm. The relative abundance of the *Ruminococcus2* also significantly decreased with scFOS (−2.10%), and significantly increased with placebo (+1.70%), resulting in a significant treatment effect. Other significant differences between arms were observed, such as an increase in *Sutterella* and *Acidaminococcus* and a decrease in *Escherichia/Shigella* and *Intestinibacter* in the scFOS arm, while the opposite effect was observed in the placebo group. However, these changes in relative abundance were limited (<2%).

**Table 5:** Change from V2 to V5 of the relative abundance of bacterial taxa in the fecal microbiota in the ITT sub-population (*n* = 30).

Taxon *Phylum* *Family* *Genus*	Arm	LS mean (%)	95%CI of LSmean	Within-arm *p*-value	Between-arm *p*-value
*Actinomycetota*	Placebo	+0.13	[−3.96, 4.21]	0.9494	**0.0485**
	scFOS	+6.03	[1.95, 10.12]	**0.0054**	
*Bifidobacteriaceae*	Placebo	+1.49	[−1.30, 4.27]	0.2835	**0.0202**
	scFOS	+6.25	[3.46, 9.03]	**0.0001**	
*Bifidobacterium*	Placebo	+1.49	[−1.30, 4.27]	0.2835	**0.0202**
	scFOS	+6.25	[3.46, 9.03]	**0.0001**	
*Pseudomonadota*				NS	NS
*Sutterellaceae*	Placebo	−1.27	[−2.50, −0.05]	**0.0427**	**0.0094**
	scFOS	+1.10	[−0.13, 2.33]	0.0764	
*Sutterella*	Placebo	−0.54	[−1.24, 0.16]	0.1265	**0.0121**
	scFOS	+0.76	[0.06, 1.47]	**0.0342**	
*Enterobacteriaceae*	Placebo	+1.37	[0.31, 2.43]	**0.0130**	**0.0126**
	scFOS	−0.58	[−1.64, 0.48]	0.2714	
*Escherichia/Shigella*	Placebo	+1.37	[0.31, 2.43]	**0.0130**	**0.0126**
	scFOS	−0.58	[−1.63, 0.48]	0.2714	
*Bacillota*				NS	NS
*Acidaminococcaceae*	Placebo	−0.76	[−1.76, 0.24]	0.1288	0. 0807
	scFOS	+0.49	[−0.51, 1.48]	0.3260	
*Acidaminococcus*	Placebo	−0.28	[−1.18, 0.62]	0.5259	**0.0255**
	scFOS	+1.19	[0.29, 2.09]	**0.0113**	
*Lachnospiraceae*				NS	NS
*Anaerostipes*	Placebo	−0.65	[−3.80, 2.49]	0.6741	**0.0159**
	scFOS	+4.94	[1.79, 8.08]	**0.0033**	
*Blautia*	Placebo	−0.56	[−5.06, 3.93]	0.8002	**0.0170**
	scFOS	−8.46	[−12.96, −3.96]	**0.0006**	
*Ruminococcus2*	Placebo	+1.70	[0.10, 3.30]	**0.0382**	**0.0020**
	scFOS	−2.10	[−3.70, −0.50]	**0.0120**	
*Peptostreptococcaceae*				NS	NS
*Intestinibacter*	Placebo	+1.76	[0.61, 2.91]	**0.0039**	**0.0170**
	scFOS	−0.27	[−1.41, 0.88]	0.6336	

Only bacterial taxa with significant differences (*p* < 0.05) between arms are presented and with variations > 1%. LS mean: mean estimated by the linear mixed model, with the 95% confidence interval (95%CI). NS, not statistically significant (*p* > 0.05). ITT, intention to treat. Bold values indicate statistically significant differences between arms (*p* < 0.05).

These bacterial changes were associated with a modification of gut fermentative activity, as highlighted by analysis of SCFA concentrations.

**Figure 4:**
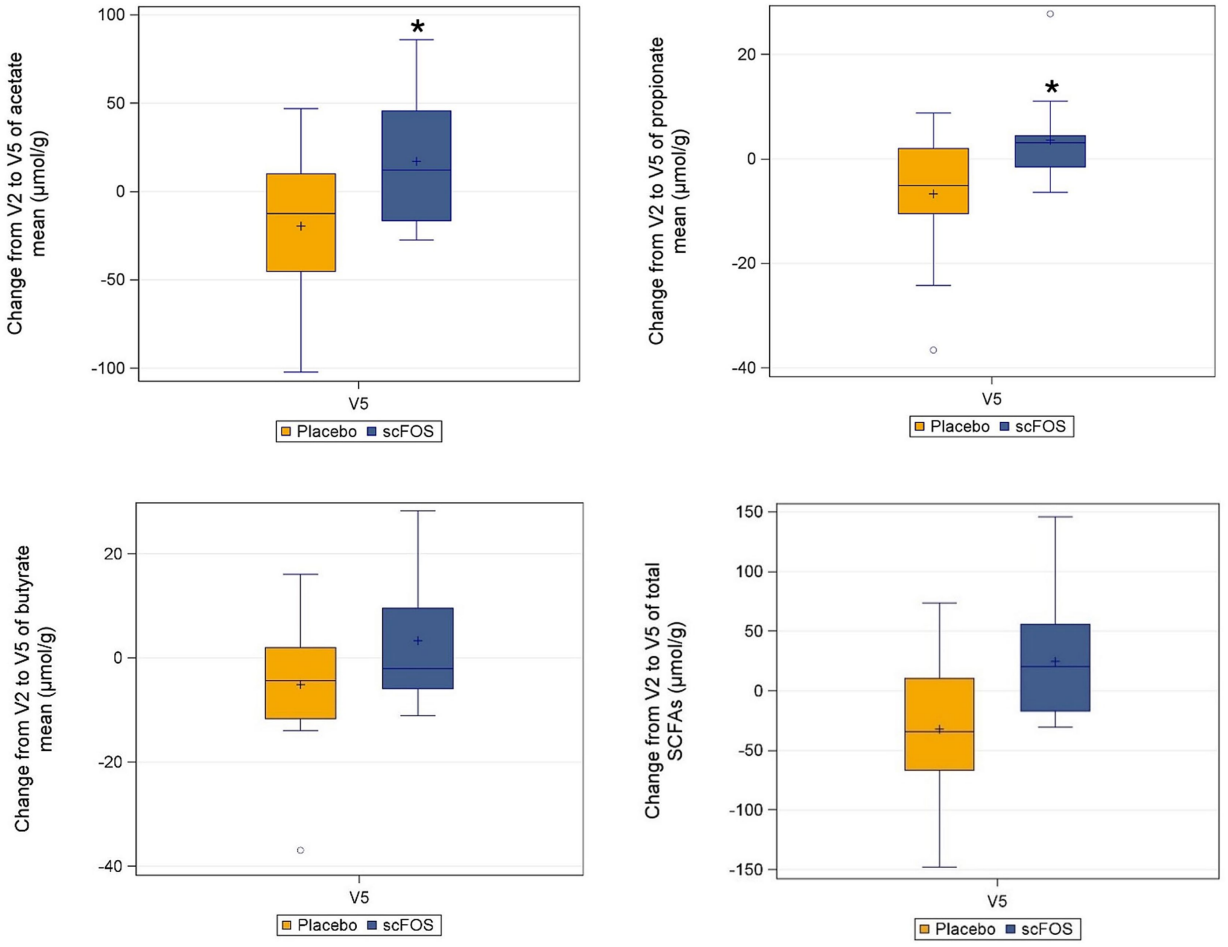
Absolute change from V2 to V5 of individual and total SCFAs per treatment arm in the ITT subpopulation (*n* = 30). *p ≤ 0.05: statistically significant difference between arms, as per the Wilcoxon-rank sum test (acetate: *p* = 0.0310; propionate: *p* = 0.0062). Butyrate: *p* = 0.1249 as per the same test. Total SCFA: *p* = 0.1234 as per the linear mixed model applied to log-transformed data.

In the ITT population, fecal acetate and propionate levels increased in the scFOS arm between V2 and V5 (median change: +12.1 μmol/g and +3.1 μmol/g respectively) whereas they decreased in the placebo arm (median change: −12.5 μmol/g and −5.1 μmol/g respectively), leading to a significant difference between arms (*p* = 0.031 and *p* = 0.0062 respectively). Conversely, the change in butyrate level did not differ significantly between treatment arms (*p* = 0.1249) (Figure 4). Regarding total SCFAs, the level tended to increase with scFOS supplementation and to decrease with placebo (mean change: +24.6 *vs* −32.2 μmol/g in the ITT population), although the treatment effect did not reach statistical significance (*p* = 0.1234 in the ITT population; *p* = 0.0886 in the PP population).

### 3.5 Safety

While the overall prevalence of adverse events in this study was relatively high (77.3% of subjects; 67.7% in the placebo arm, 85.7% in the scFOS arm), most were expected gastrointestinal symptoms consistent with the known safety profile of the investigational product scFOS. These events were predominantly mild or moderate, including flatulence, bloating and abdominal discomfort, commonly associated with high-dose fermentable fiber intake. Importantly, no serious adverse effects were reported. Overall, no safety concerns were raised following the use of Actilight^®^ 950P, 2 sachets of 10 g per day, for a period of 12 weeks.

## 4 Discussion

In recent years, growing attention has been directed toward the role of the gut microbiota in the etiology and pathophysiology of metabolic disorders such as obesity and T2D. Nutritional strategies, particularly those involving prebiotic fibers, are increasingly recognized as promising tools to modulate the gut microbiota and, in turn, influence host metabolic health.

Systematic reviews and meta-analyses have highlighted the potential of prebiotics to modulate glucose metabolism, particularly in individuals with obesity or T2D. However, findings remain conflicting, probably due to variability in the study populations’ health status, diet and lifestyle habits, type and dose of prebiotic, and intervention duration (15, 16). In obese non-diabetic individuals, inulin supplementation at 10 g/day for 12 weeks, whether or not combined with caloric restriction, has generally shown no clear benefit on glucose homeostasis (26–28), as their glycemic parameters are almost normal. In contrast, more pronounced effects have been observed in individuals with T2D (25, 29–32), likely due to their altered metabolic status, which may make them more responsive to interventions targeting glucose regulation.

Although we observed here that a 12-week scFOS supplementation led to beneficial changes in gut microbiota composition and fermentative activity in overweight individuals with prediabetes, it did not result in significant improvements in glucose metabolism parameters in this population at risk of developing T2D. The absence of effect on HbA1c is consistent with findings from a study in similar prediabetic individuals supplemented with 15 g/day of inulin for 6 months (33). Reductions in fasting insulin and HOMA-IR were reported, but these changes remain to be confirmed as there was no control arm in that longer term study. In addition, a higher dose of inulin (30 g/day) administered for only 6 weeks to subjects with prediabetes did not yield significant changes in fasting metabolic markers as compared to placebo (34).

In our study, the lack of significant effects on the primary outcome HbA1c and other metabolic markers may be partly explained by almost normal baseline values, which could have limited the potential for measurable improvement. The initial assumption of a 0.8% reduction proved optimistic, and the trial may have been underpowered to detect smaller, yet potentially meaningful, changes. Indeed, the observed evolution of glucose homeostasis parameters suggests a positive treatment effect, which might have reached statistical significance with a larger sample size or in a population with more pronounced metabolic impairments, as seen in previous studies involving T2D patients.

Regarding anthropometric secondary outcomes, scFOS supplementation was associated with a modest but favorable shift in body composition, characterized by a reduction in fat mass and a concurrent increase in lean mass. While body weight and BMI tended to increase in the placebo arm, they remained relatively stable in the scFOS arm.

Evidence from prediabetic populations remains limited in the literature regarding those parameters. One study reported a reduction in body weight following 30 g/day of inulin for 6 weeks (34), and a decrease in fat mass was observed when inulin supplementation was extended to 18 weeks and combined with a weight loss program (35).

In obese individuals, inulin supplementation at 16 g/day for 12 weeks did not significantly affect anthropometric or metabolic parameters (26). When combined with caloric restriction, results are conflicting, with some studies reporting modest reductions in body weight and BMI (27, 36) while others found no significant effects (27, 37). More consistent improvements have been observed in T2D populations (25, 29, 32).

To our knowledge, this is the first study to report a modest reduction in fat mass following 12 weeks of scFOS supplementation at 20 g/day in overweight prediabetic individuals, without any concurrent caloric restriction or structured weight loss program. This observed reduction in fat mass does not appear to be driven by changes in food intake, as no statistically significant differences were detected between arms in appetite scores (hunger and satiety assessed during 4 h after the meal; data not shown), total energy, macronutrient or fiber intake (apart from scFOS; Supplementary Table 1), nor in physical activity levels (recorded qualitatively at each visit). We therefore speculate, based on prior evidence, that other mechanisms, such as microbiota modulation or SCFA-mediated metabolic effects, may have contributed to the changes in body composition.

Importantly, our study concurrently assessed gut microbiota composition as secondary outcome, alongside metabolic and anthropometric outcomes. We demonstrated that scFOS supplementation selectively promoted the growth of certain bacterial taxa while reducing others in a subpopulation of 30 overweight individuals with prediabetes. Several studies have highlighted differences in gut microbiota composition between healthy individuals and those with metabolic disorders (2–4, 30, 38), suggesting that specific microbial signatures may be linked to metabolic health.

In this study, we observed a significant reduction in *α*-diversity indices in the scFOS arm, pointing out a decrease in the observed number of taxa, but also in their evenness, which may reflect the selective enrichment of specific bacterial taxa at the expense of others. A similar decrease in microbial diversity has been reported in prediabetic individuals supplemented with inulin (33). We cannot further interpret this reduction in diversity in the absence of complementary functional measures that would allow us to characterize the physiological impact of these ecological changes. At the same time, we observed a higher *β*-diversity in the scFOS arm than in the placebo arm, indicating a higher dissimilarity between V2 and V5 samples. Although not statistically significant, this trend may highlight changes in the qualitative and quantitative composition of microbiota due to the prebiotic: some new species were found at the end of supplementation (or became detectable) and some relative abundances were modified.

At the phylum level, scFOS supplementation resulted in a marked increase in the relative abundance of *Actinomycetota* (previously named *Actinobacteria*), primarily driven by the proliferation of the *Bifidobacterium* genus (+6.25%). This bifidogenic effect is well-documented in the literature and has been consistently observed across various populations and doses of scFOS supplementation ranging from 1 to 20 g/day (39–44). Other prebiotic fibers have also been shown to increase *Bifidobacterium* relative abundance in individuals with obesity, prediabetes or diabetes, as recently reviewed by Alonso-Allende et al. (15).

Beyond this well-established bifidogenic effect, scFOS supplementation induced additional significant shifts in microbial composition. Notably, we observed an increase in the relative abundance of *Anaerostipes* (+5%). The enrichment of this genus has also been reported following prebiotic interventions in obese (28, 37), prediabetic (33), and diabetic individuals (30). These two genera, *Bifidobacterium* and *Anaerostipes,* are typically found in higher abundance in healthy individuals compared to those with obesity, prediabetes, or T2D (3, 4, 30). *Bifidobacterium* species are early colonizers of the human gut and are known to confer intestinal, immune, and metabolic benefits throughout life (45). A study has shown that individuals with higher visceral fat exhibited a lower relative abundance of *Bifidobacterium*, with a significant inverse correlation observed between *Bifidobacterium* level and visceral fat (46). This observation aligns with our findings, which revealed a substantial increase in *Bifidobacterium* abundance and a slight reduction in fat mass in the scFOS group compared to the placebo. Additionally, *Anaerostipes*, a butyrate-producing genus, is commonly associated with a healthy gut profile and reduced risk of metabolic disease (47, 48).

Conversely, scFOS supplementation led to a reduction in the relative abundance of *Blautia* (−8.5%) and *Ruminococcus2* (−2.1%), two phylogenetically close genera (49). The *Blautia* genus has been found in higher abundance in individuals with prediabetes (2) and positively correlated with glycemic and anthropometric markers such as fasting glucose, HOMA-IR, HbA1c and waist circumference (2, 38). While *Blautia* has been associated with both healthy and unhealthy phenotypes depending on the context (2–4, 30, 50, 51), its reduction in our study may reflect a shift toward a healthier microbial profile. The depletion of *Ruminococcus2* has also been reported following a mixed-fiber dietary intervention (52). Moreover, *Ruminococcus2* has been observed as more prevalent in patients with severe fatty liver disease and hyperlipidemia (53) and as positively correlated with indicators of bodyweight (including waistline and body mass index) and serum lipids (including low density lipoprotein, triglyceride and total cholesterol levels) (54). This suggests that the decrease of *Ruminococccus2* with scFOS supplementation may be associated with improved metabolic health and body composition.

We also observed a slight (but statistically significant) increase in *Sutterella* after scFOS supplementation. This genus is reported to be reduced in individuals with T2D and obesity (38, 51, 55, 56), although findings remain inconsistent (2, 4). Notably, *Sutterella* abundance was negatively associated with HbA1c and fasting glucose blood levels in a recent study comparing obese individuals with and without T2D (38) and found in lower abundance in women with a higher visceral fat area (46), suggesting a potential role in glucose homeostasis and body composition. Finally, the increase of *Catenibacterium* abundance in the scFOS group aligns with previous observations following inulin supplementation in obese individuals undergoing caloric restriction (28, 37). However, the role of *Catenibacterium* remains controversial, with conflicting findings across studies (30, 57).

These bacterial compositional changes were accompanied by enhanced microbial fermentative activity, as evidenced by increased levels of fecal acetate and propionate in the scFOS arm, whereas these metabolites decreased in the placebo arm, with a statistically significant product effect. We speculate that the lack of a statistically significant difference between arms in fecal butyrate, despite a marked rise in *Anaerostipes* in the scFOS arm, a known butyrate-producing genus, may be explained by the rapid absorption of butyrate by colonocytes, which use it as a primary energy source. Additionally, the observed reduction in *Blautia*, a genus that includes butyrate-producing species, may have counterbalanced the increased production from *Anaerostipes*, resulting in almost no net change in fecal butyrate levels. It is worth noting that fecal SCFAs are indicative of colonic microbial fermentation activity and reflect only a portion of total SCFA production, as the majority are absorbed in the colon and utilized by host tissues where they can exert metabolic effects.

The increase in acetate and propionate contrasts with some studies in obese individuals supplemented with oligofructose-inulin (37, 58) but aligns with results from a recent study in individuals with T2D consuming prebiotics (30). This finding may contribute to fat mass reduction in scFOS-supplemented subjects through mechanisms involving energy metabolism, lipid storage, and inflammation regulation (7).

Our study has several limitations that should be acknowledged. First, the primary endpoint assumption regarding HbA1c change from V2 to V5 was not met, with an expected difference between groups of −0.8% contrasting with a mean observed difference in change of +0.025%, resulting in an underpowered study. Secondly, a potential effect of maltodextrin (chosen as a placebo) on clinical endpoints cannot be excluded and may have influenced the observed outcomes. Third, most baseline biomarkers were within normal ranges, potentially limiting the magnitude of detectable changes. Fourth, although age and sex are known as strong predictors of metabolic and body composition (59), the analyses presented here do not include them as covariates, as models including them did not substantially modify the results, nor did they lead to different conclusions in terms of statistical significance, while being less reliable (data not shown). Finally, microbiota analyses were conducted on a limited subset of participants, and although the results are consistent with previous findings, they should be considered preliminary and interpreted with caution until confirmed in larger, adequately powered studies. The analyses also concerned a large number of taxa, and no *p*-value adjustment was applied to control for the multiplicity of tests.

## 5 Conclusion

Altogether, although no significant effects were observed on the primary outcome HbA1c and other glycemic markers within the 12-week study period, our findings suggest that scFOS supplementation in overweight individuals with prediabetes induces favorable shifts in gut microbiota composition, including the enrichment of health-associated bacterial taxa such as *Bifidobacterium* and *Anaerostipes*. These microbial changes were accompanied by modest improvements in body composition, notably a reduction of fat mass. The observed modulation of the gut microbiota due to scFOS supports the hypothesis that targeted nutritional interventions can help restore microbial balance in at-risk populations. While the metabolic benefits of scFOS appeared limited in this cohort exhibiting a close-to-normal metabolic pattern, the reduction in fat mass and the microbial signatures associated with improved metabolic health remain promising. These results underscore the potential of scFOS prebiotic supplementation as a preventive strategy to address excess body weight and fat accumulation, key contributors to the development of obesity and T2D. By improving gut microbiota composition, such interventions may contribute to broader efforts aimed at mitigating the global burden of metabolic diseases. Further research in larger and more metabolically impaired populations is warranted to confirm these findings and assess their clinical relevance for the prevention of metabolic diseases.

## Data Availability

The original contributions presented in the study are publicly available. The raw 16S rRNA gene amplicon sequencing data can be found in the European Nucleotide Archive (ENA) under accession number PRJEB104945 (https://www.ebi.ac.uk/ena/browser/view/PRJEB104945).

## Footnotes

1
https://www.clinicaltrials.gov/study/NCT04767672
