# Supplementary material for: Targeting gut microbiota with short-chain fructo-oligosaccharides prebiotic fibers to support metabolic health in overweight prediabetic adults: a randomized, double-blinded, placebo-controlled study
Source: Front Nutr. 2025 Dec 17;12:1718169. doi: 10.3389/fnut.2025.1718169 (PMC12753434; doi:10.3389/fnut.2025.1718169)
Supplement: Supplementary file 1 [file Table_1.docx]

**Supplementary Table 1: Absolute change from V2 to V5 of total energy, fat, carbohydrate, protein and fiber intake per treatment arm in the ITT population**

| **Variable** | **Placebo (n=31)** | **scFOS (n=35)** | **P-value** |
| --- | --- | --- | --- |
| **Total energy intake (kcal/day)** |  |  |  |
| N | 27 | 32 |  |
| Median (Q1; Q3) | 42.0 (-325.0; 329.0) | 101.5 (-159.0; 361.0) | 1.0000 |
| **Fat intake (g/day)** | |  |  |
| N | 27 | 32 |  |
| Median (Q1; Q3) | -5.2 (-22.1; 10.3) | 3.9 (-15.8; 13.0) | 1.0000 |
| **Carbohydrate intake (g/day)** |  |  |  |
| N | 27 | 32 |  |
| Median (Q1; Q3) | 2.0 (-31.0; 45.0) | 13.3 (-21.5; 48.0) | 1.0000 |
| **Protein intake (g/day)** |  |  |  |
| N | 27 | 32 |  |
| Median (Q1; Q3) | 2.7 (-18.7; 12.6) | -2.9 (-10.5; 11.5) | 1.0000 |
| **Fiber intake (g/day)** |  |  |  |
| N | 27 | 32 |  |
| Median (Q1; Q3) | 1.4 (-1.1; 4.0) | 2.8 (-1.6; 5.7) | 0.9026 |

*Values are medians of the difference V5-V2 calculated for each subject, with the between-arm p-value obtained from the Wilcoxon-rank sum test. P-values were adjusted due to the multiplicity of comparisons (multiple visits, not displayed here; Bonferroni-Holm method).*
